# Preparation and Specific Capacitance Properties of Sulfur, Nitrogen Co-Doped Graphene Quantum Dots

**DOI:** 10.1186/s11671-019-3045-4

**Published:** 2019-07-01

**Authors:** Zhong Ouyang, Yun Lei, Yunpeng Chen, Zheng Zhang, Zicong Jiang, Jiaxin Hu, Yuanyuan Lin

**Affiliations:** School of Resources and Environmental Engineering, Wuhan University of Technology, Luoshi Road 122, Wuhan, 430070 Hubei China

**Keywords:** Graphene quantum dots, Top-down, Doping ratios, The specific capacitance

## Abstract

Sulfur, nitrogen co-doped graphene quantum dots (S, N-GQDs) with high crystallinity were obtained by a top-down strategy. The as-prepared S, N-GQDs were investigated and the results indicate that S, N-GQDs exhibit a transverse dimension about 20 nm and a topographic height of 1–2 layers graphene. The incorporation of S, N can effectively reduce the layers of GQDs and strip the graphene sheets. Moreover, the S, N-GQDs reveal an absorption band located at 405 nm and exhibit an adjustable fluorescence characteristic in the excitation-visible range. Meanwhile, the S, N-GQDs shows a high specific capacitance of 362.60 F g^−1^ at a fixed scan rate of 5 mV s ^−1^. This high performance is ascribed to the additional high pseudocapacitance provided by the doped S, N and the doping state acting as a trap state to enhance the charge storage capacity. The high specific capacitance advantages of S, N-GQDs illustrate their potential prospects in the capacitors.

## Introduction

Graphene quantum dots (GQDs) have caused a lot of attention due to their good biocompatibility, chemical inertia, photoluminescence, upconversion luminescence, and widely used in bioimaging, optoelectronic devices, photocatalysis, biosensors, fuel cells, and heavy metal ion detection [1–4]. Up till now, GQDs have been obtained through various synthetic methods involving “bottom-up” and “top-down” [5]. The bottom-up method includes converting a suitable precursor to GQDs by pyrolysis or carbonization, stepwise organic synthesis, cage-opening of C_60_, and the like [6–9]. On the contrary, the “top-down” method is to cut large-sized carbon materials into nanosized carbon nanoparticles by physical or chemical methods, including electron beam lithography, acid stripping, electrochemical oxidation, hydrothermal synthesis, etc. [10–13]. Compared with the bottom-up methods, the top-down routes have a great deal of advantages including wide resources, large production, and easy preparation. In addition, GQDs prepared by the top-down approaches typically have oxygen-containing functional groups on the edges, so as to promote their dissolution, functionalization, and passivation [14].

Doping acts as an effective method to regulate the properties of nanomaterials. The n-type and p-type doping of the semiconductor can change the electronic structure of the semiconductor material, which causes changes in optical and electrical properties [15–17]. Doped GQDs draw on the concept of semiconductor doping, which mainly refers to the introduction of S, N, Se, and other elements into defective GQDs consisting of C and O elements [18–21]. The N atom has the same atom size as C atom and five valence electrons. N bonded with C atom has been extensively applied in chemical doping of carbon nanomaterials [22, 23]. Hu et al. synthesized highly blue-luminescent N-GQDs that showed bright luminescence and excellent biocompatibility [24]. Majumder et al. prepared N-GQDs/ZnO nanorod with superior photoconversion efficiency and better photoelectrochemical properties [25]. Yan et al. constructed a novel N-GQDs-ZnNb_2_O_6_/g-C_3_N_4_ catalysts, which exhibited a much higher hydrogen-evolution rate [26]. Chen et al. obtained N-GQDs/Bi_2_O_3_ catalyst for electrochemical reduction of CO_2_ [27]. Recently, some researchers have successfully prepared S, N co-doped GQDs that showed excellent performance. Zhang et al. employed a one-step hydrothermal method to attain SN-GQDs with brighter luminescence [28]. Xu et al. fabricated S, N co-doped GQDs with tunable luminescence, which showed highly selective and sensitive fluorescent detection of Fe^3+^ [29]. Mondal et al. used S, N co-doped GQDs as an excellent sensor for nitro explosives [30]. Zheng et al. developed SN-GQD/TiO_2_ photocatalyst that exhibited 3.2 times H_2_O_2_ yield than bare TiO_2_ [31]. Though there are some reported work on the optical and sensing properties of the sulfur, nitrogen co-doped graphene quantum dots (S, N-GQDs), the influence of S and N doping on the specific capacitance characteristics of S, N-GQDs are rarely studied.

In this paper, we reported a top-down hydrothermal method to synthesize S, N co-doped GQDs (S, N-GQDs) via using graphite as the C source and thiourea as S and N sources. At the same time, the influence of doping ratios on the electrochemical properties of S, N-GQDs were investigated by changing the ratio of thiourea from 1:1 to 1:3.

## Methods and Experimental

### The Aims of the Study

In order to study the effect of doping ratio on the specific capacitance performance of S, N-GQDs, different doping ratios S, N-GQDs were prepared by a simple top-down hydrothermal method. To initially evaluate its specific capacitance characteristics, the specific capacitance of different doping ratios S, N-GQDs was measured by cyclic voltammetry.

### Materials

Graphite (99.9%), sulfuric acid (H_2_SO_4_), nitric acid (HNO_3_), hydrogen peroxide (H_2_O_2_), sodium nitrate (NaNO_3_), anhydrous ethanol (CH_3_CH_2_OH), thiourea (CH_4_N_2_S), anhydrous sodium sulfite (Na_2_SO_3_), potassium permanganate (KMnO_4_), and sodium hydroxide (NaOH). All materials were used of analytically pure and without any further purification.

### Preparation of Graphite Oxide

Graphite oxide were obtained by a typical Hummers method. Firstly, 5 g of flake graphite was mixed with 110 ml H_2_SO_4_, 2.5 g NaNO_3_ and 15 g KMnO_4_, and the mixture was stirred at 6 °C for 90 min. Then, the mixture was stirred at 35–40 °C for 30 min to further oxidize the graphite. Finally, 220 ml of DI water was added to the solution and reacted at 90–100 °C for 15 min, and 30 ml of H_2_O_2_ (30%) was added.

### Synthesis of S, N-GQDs

At first, graphene (100 mg) obtained by thermal reduction of graphite oxide was added to a mixture of H_2_SO_4_ (60 ml) and HNO_3_ (20 ml). The solution was sonicated for 10 h and washed by centrifugation to remove excess acid. Secondly, the product was respectively dispersed with 100 mg, 200 mg, 300 mg of thiourea (the mass ratio of graphene to thiourea was 1:1, 1:2, and 1:3, respectively) in 80 ml of deionized water, and the pH value was adjusted to 8.0 with 0.1 mol L^−1^ NaOH solution. The graphene oxide suspension was transferred to an autoclave and reacted at 200 °C for 10 h. Lastly, the suspension was filtered by a 0.22 μm micropore filter, and the filtrate was dialyzed in a dialysis bag for 24 h to obtain S, N-GQDs. The different doping ratios were denoted as S, N-GQDs-1 (1:1), S, N-GQDs-2 (1:2), and S, N-GQDs-3 (1:3), respectively.

### Characterization

The morphology of GQDs and S, N-GQDs was examined by an atomic force microscope (AFM) (Multimode 8) and a TEM (JEM-2100F). The FTIR spectrum was obtained by a Nicolet iS10 spectrometer. X-ray photoelectron spectroscopy (XPS) was obtained on an ESCALAB 250XI electron spectrometer. UV-visible spectra were analyzed by UV5500 spectrophotometer. Photoluminescence (PL) spectra were characterized on a Cary Eclipse fluorescence spectrophotometer.

### Electrochemical Measurement

Cyclic voltammetry (CV) were performed at an electrochemical working station (CHI650E). In the three-electrode system, Pt electrode, calomel electrode, and glassy carbon electrode were respectively used as the counter electrode, reference electrode, and working electrode. CV measurements were recorded at a scanning rata from 5 to 200 mV s^−1^ in 2 M KOH solution. The specific capacitance (C) of S, N-GQDs can be evaluated by using Eq. (1):

1 $$ C=\frac{\int IdV}{vm\Delta  V} $$

where ∫*IdV* is the area surrounded by CV curve, *∆V*(V) is the voltage window, *v* (mV s^−1^) is the scan rate, and *m*(g) is the mass of the S, N-GQDs in the working electrode.

## Results and Discussion

### Morphology Analysis

**Fig. 1:**
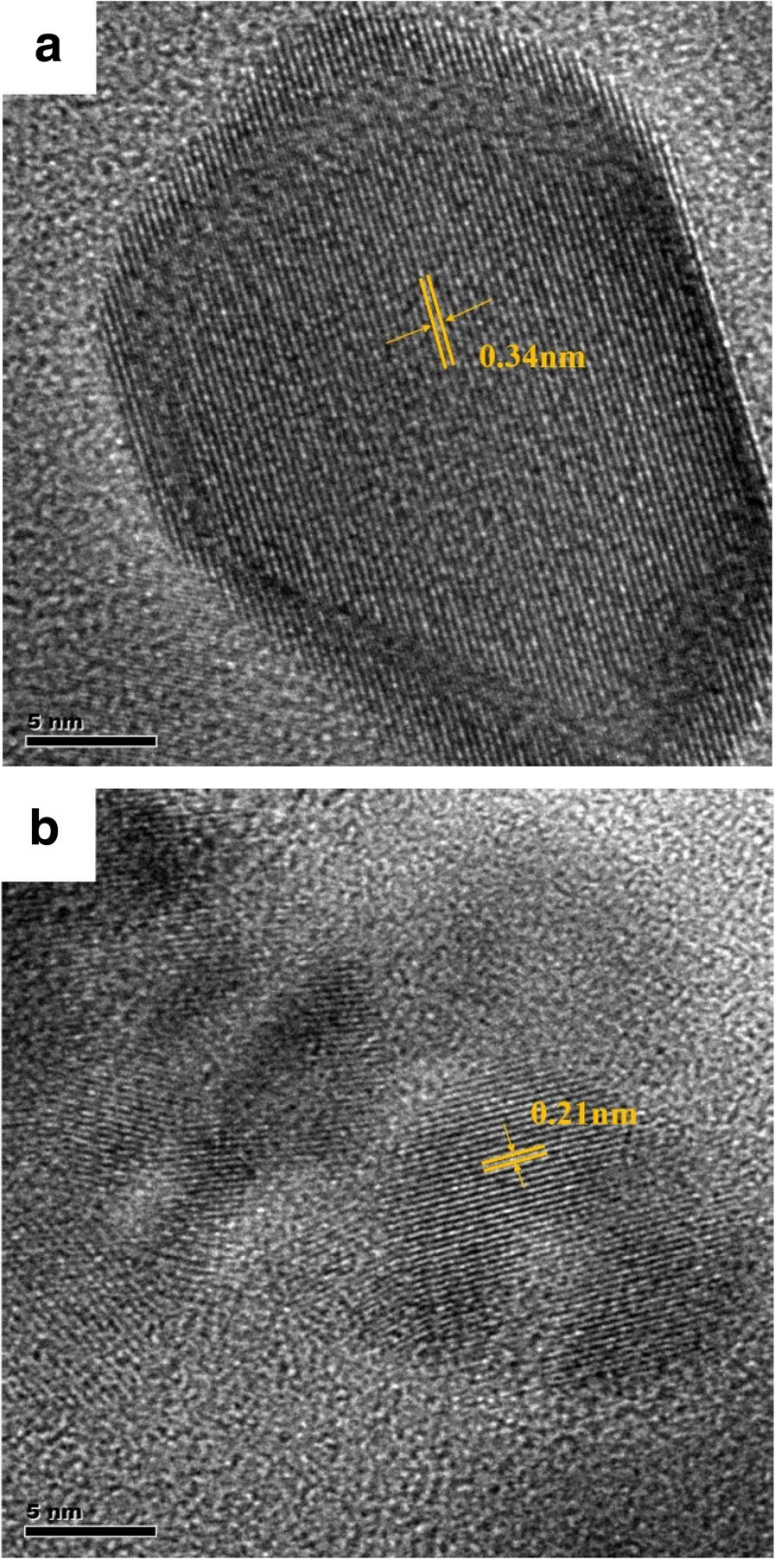
HRTEM image of **a** S, N-GQDs and **b** GQDs

The high-resolution transmission electron microscope (HRTEM) image of S, N-GQDs and GQDs were shown in Fig. 1. S, N-GQDs have a size of about 20 nm and present high crystallization with an interplanar distance of 0.34 nm, which corresponds to the (002) crystal face of graphene [32]. Figure 1b shows that the lateral size of GQDs is about 10 nm, and the lattice spacing of GQDs is measured to be 0.21 nm, which belongs to in-plane (100) facet of graphene [33]. The results illustrate that S, N-GQDs may be made up of nanocrystalline cores of graphitic sp^2^ C atoms and the incorporation of S, N has no effect on the lattice structure of GQDs [34].

**Fig. 2:**
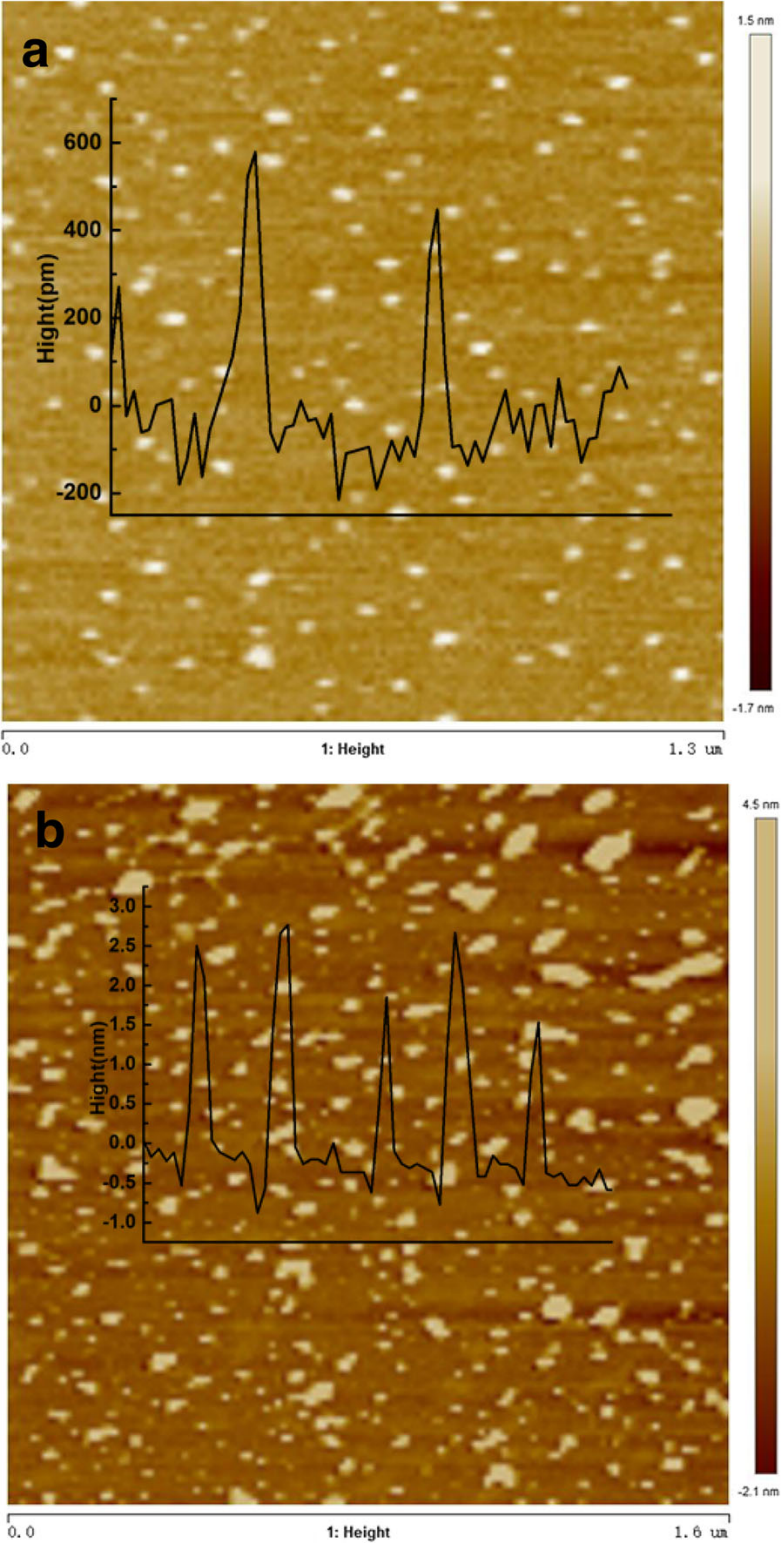
AFM image of **a** S, N-GQDs and **b** GQDs

As shown in Fig. 2, the topography of S, N-GQDs and GQDs were investigated by AFM. The as-prepared S, N-GQDs and GQDs show uniform size distribution. In Fig. 2a, the average height of S, N-GQDs is about 0.5 nm, indicating that S, N-GQDs have about 1–2 layers graphene. Figure 2b shows that the average thickness of the GQDs prepared by the hydrothermal method is about 1.5 to 2.5 nm, suggesting that GQDs have about 4–6 layers graphene. Comparing the thickness of both S, N-GQDs and GQDs, the former is significantly reduced. The results demonstrate that the incorporation of S, N can effectively reduce the layers of GQDs and strip the graphene sheets.

### FT-IR Analysis

**Fig. 3:**
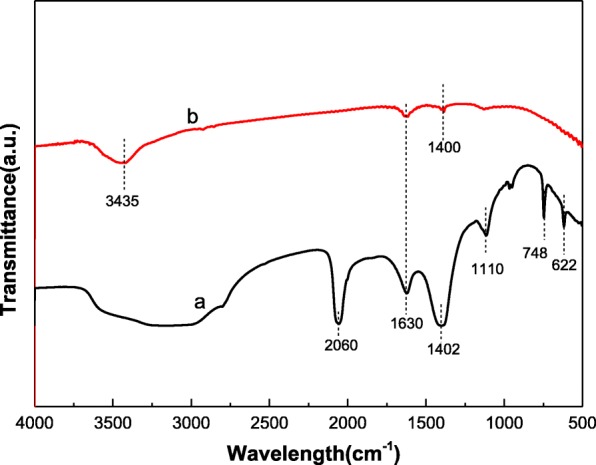
FTIR spectra of **a** S, N-GQDs and **b** GQDs

Figure 3 shows the FT-IR spectra of S, N-GQDs and GQDs. For S, N-GQDs (Fig. 3a), the bands at 2060 cm^−1^, 1402 cm^−1^, and 1110 cm^−1^ are related to the vibrational absorption band of C ≡ N, C-N, and C=S, respectively [34, 35]. The absorption peaks at 748 cm^−1^ and 622 cm^−1^ correspond to C-S stretching vibrations [34, 36]. As can be seen from Fig. 3b, GQDs have weaker absorption peaks. The absorption peaks at 3435 cm^−1^, 1630 cm^−1^, and 1400 cm^−1^ are the O-H stretching vibration of water in the air, the stretching vibration of the C=C bond in the graphite structure and the stretching vibration of C-H, respectively [21]. The FT-IR results show that S and N can be successfully doped into GQDs by the hydrothermal method.

### XPS Analysis

**Fig. 4:**
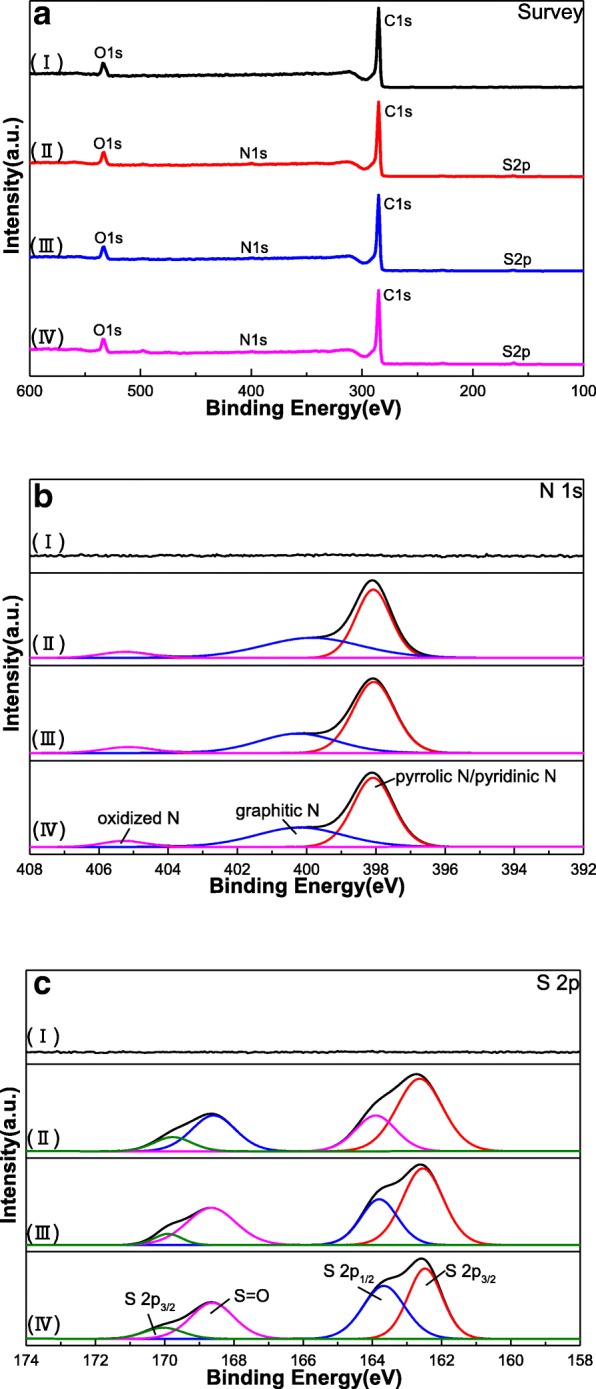
XPS spectra of the as-prepared samples: **a** survey spectra, **b** and **c** high resolution of N 1 s, and S 2p, respectively. (I) GQDs, (II) S, N-GQDs-1, (III) S, N-GQDs-2, and (IV) S, N-GQDs-3

In order to reveal the chemical states and elemental compositions of the as-prepared S, N-GQDs with different doping ratios and GQDs, XPS measurement was employed as shown in Fig. 4. The full scan XPS spectrum of GQDs (Fig. 4a) exhibits two peaks at 534 and 285 eV, which correspond to O 1s and C 1s. Additional peaks of 398 and 163 eV appeared in the full spectrum of S, N-GQDs with different doping ratios are attributed to N 1s and S 2p. The high-resolution N 1s XPS spectrum of S, N-GQDs with different doping ratios (Fig. 4b) shows three peaks around 398.10 eV, 400.20 eV, and 405.20 eV are attributed to pyrrolic N (C–N–C) or pyridinic N, graphitic N, and oxidized N, respectively [34, 37]. Figure 4c shows the high-resolution S 2p XPS spectrum of S, N-GQDs with different doping ratios and peaks located at 162.4 eV, 163.6 eV, 168.6 eV, and 170.2 eV, corresponding to S 2p_3/2_, S 2p_1/2_, S=O, and S 2p_3/2_, respectively [34]. As we all know, S and N doping are beneficial for improving the electrochemical properties of materials [38, 39]. Since the S and the N atoms are incorporated into the graphene layer, the carbon atoms in the plane are replaced and more electrons are supplied to the π-conjugated system of the graphene, thereby improving the conductivity of the samples [40]. In addition, the presence of S and N atoms in the graphene structure can provide an electrochemically active site and pseudocapacitance effects to enhance the capacitive properties of the material [41, 42]. These results confirm that the successful sulfur and nitrogen were doped into the framework of GQDs, which are consistent with the result shown in FT-IR.

### UV-Visible Analysis

**Fig. 5:**
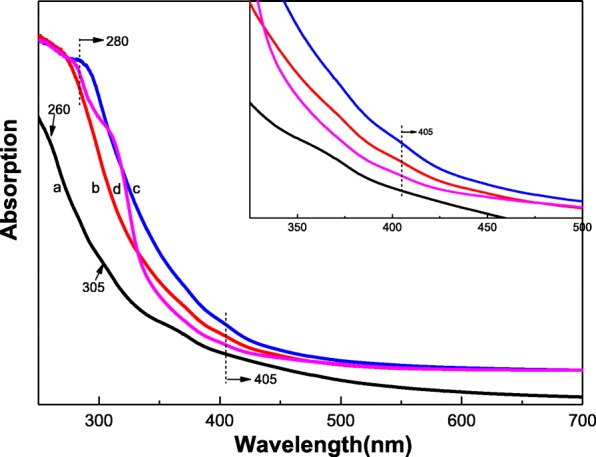
UV-visible spectra of **a** GQDs, **b** S, N-GQDs-1, **c** S, N-GQDs-2, and **d** S, N-GQDs-3

Figure 5 shows the UV-visible spectra of GQDs and S, N-GQDs with different doping ratios. In Fig. 5a, the absorption peak located at 260 nm and 305 nm can be attributed to the π-π* transition of C=C and the n-π* transition of the C=O bond, respectively [34]. Figure 5b–d shows the UV-visible spectra of S, N-GQDs-1, S, N-GQDs-2, and S, N-GQDs-3, respectively. Compared with the π-π*, transition absorption peak position of C=C in GQDs, S, N-GQDs have obvious “red shift” phenomenon. Moreover, S, N-GQDs have characteristic peaks around 405 nm corresponds to the n-π* transition of the conjugated C=N [43], which may be attributed to changing the surface state of GQDs with the incorporation of N. There are no S-related peaks around 550 and 595 nm because the electronegativity of S and C is so close that the difference between the two energy levels is negligible [44]. This result indicates that the doping of S and N can effectively improve the absorption of visible light by GQDs.

### PL Analysis

**Fig. 6:**
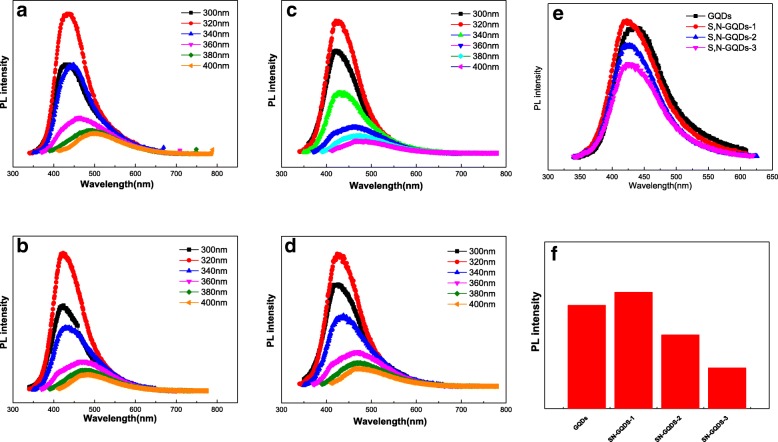
PL spectra of **a** GQDs, **b** S, N-GQDs-1, **c** S, N-GQDs-2, and **d** S, N-GQDs-3 under different excitation light; **e** PL spectra of GQDs and different doping ratio of S, N-GQDs at 320 nm excitation; **f** the trend of PL intensity variation of GQDs and different doping ratios S and N-GQDs at 320 nm excitation

The photoluminescence (PL) spectra of GQDs and S, N-GQDs solutions under different excitation wavelengths are shown in Fig. 6a–d. The spectra show that as the excitation wavelength increases from 300 to 400 nm, the PL intensity of GQDs and S, N-GQDs both increase first and then decrease. At the excitation wavelength of 320 nm, the PL intensity of the GQDs reaches a maximum, and the PL peak is located at 430 nm, corresponding to the π* → n transition of the carbonyl or carboxyl group [45]. Moreover, as the excitation wavelength increases from 300 to 400 nm, the positions of the PL spectral emission peaks of S, N-GQDs gradually red-shifted, indicating that the hydrothermally synthesized doped GQDs exhibit excitation wavelength dependence fluorescence characteristics. It is found that S, N-GQDs exhibit photoemission related to excitation wavelength due to changes in particle size distribution and impurity states [46–48]. The size change of S, N-GQDs produces discrete sp^2^-related localized states at the LUMO and HOMO levels [49]. The electronic transition from these localized states are responsible for the red shift of the PL emission peak position. On the other hand, different functional groups at the edges of the S, N-GQDs such as oxygen and nitrogen atoms or S=O may create a trap state between the LUMO and HOMO levels, which also exhibits excitation wavelength dependence photoemission [49]. The PL spectra of GQDs and S, N-GQDs at 320 nm excitation wavelength are shown in Fig. 6e. Compared with undoped samples, the sulfur and nitrogen-doped samples show significant shifts in peak position when excited at 320 nm, which can be attributed to the strong electron affinity of S and N in S, N-GQDs [50]. Figure 6f shows a comparison of PL intensity for GQDs and S, N-GQDs with different doping ratios at 320 nm excitation. The result shows that S, N-GQDs-1 exhibits the best PL intensity.

### Specific Capacitance Performance Analysis

**Fig. 7:**
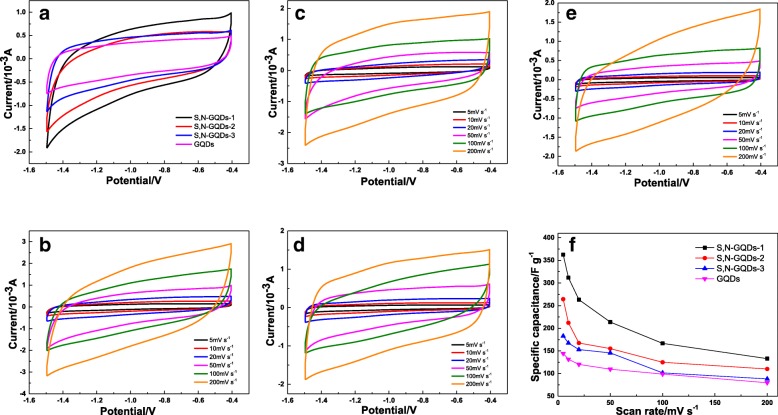
CV curves of S, N-GQDs with different doping ratios at a scan rate of 50 mV/s (**a**). CV curves of S, N-GQDs-1 (**b**), S, N-GQDs-2 (**c**), S, N-GQDs-3 (**d**), and GQDs (**e**) at different scan rates of 5, 10, 20, 50, 100, and 200 mV/s. **f** Specific capacitance values of S, N-GQDs-1, S, N-GQDs-2, S, N-GQDs-3, and GQDs at different scan rates of 5, 10, 20, 50, 100, and 200 mV s^−1^

The specific capacitance of S, N-GQDs with different doping ratios was evaluated by cyclic voltammetry (CV). Figure 7a presents an approximate rectangular shape of CV curves at the scanning rate of 50 mV s^−1^, indicating a remarkably capacitive behavior. It is noteworthy that the feature of curves is nearly symmetrical without obvious redox peaks, which indicates a typical characteristic of high inevitability electric double-layer capacitance (EDLC) behavior [51]. The CV curves of the S, N-GQDs-1, S, N-GQDs-2, S, N-GQDs-3, and GQDs with different scan rates (5–200 mV s^−1^) are shown in Fig. 7b–e, which indicates a fast voltage inversion current response and low ion transmission resistance in the electrode.

The specific capacitance values of samples with different scanning rates calculated by Eq. (1) are shown in Table 1. S, N-GQDs have a larger area than GQDs, indicating a larger specific capacitance value, which attributed to the additional high pseudocapacitance provided by the doped S, N and the doping state acting as a trap state helps to enhance the charge storage capacity [52, 53]. The S, N-GQDs-1 shows excellent capacitive behavior with the specific capacitance of 362.60 F g^−1^ at a fixed scan rate of 5 mV s^−1^. However, S, N-GQDs-2, S, N-GQDs-3 with higher S, N contents show lower specific capacitance values because higher S, N content leads to more oxygen vacancies. These oxygen vacancies fill the impurity state, thereby suppressing the specific capacitance behavior [35]. Therefore, S, N-GQDs-1 is more suitable for application on energy storage devices. In addition, the specific capacitance values of the prepared S, N-GQDs and reported graphene-based materials or other nanomaterials are listed in Table 2. Obviously, S, N-GQDs exhibit superior performance compared to other materials. Figure 7f shows a comparison of capacitance values when the scanning rates is changed from 5 to 200 mV s^−1^. For S, N-GQDs and GQDs at a lower scanning rate, higher specific capacitance and lower current density can be attributed to the internal resistance of the electrode. As the scanning rate is increased, the ions are confined to the external surface of the electrode, which leads to a decrease of specific capacitance. The lower current density causes ions to penetrate the internal structure of the electrode material, which facilitates the capacitive behavior. While the scanning rate is decreased, the lower current density promotes the ions to penetrate into the internal structure of the electrode, which facilitates the capacitive behavior [68].

**Table 1:** Specific capacitance values (F g^−1^) calculated from CV curves at the scan rate ranging from 5 to 200 mV s^−1^

Sample	Specific capacitance at different scan rate (mV s^−1^)					
	5	10	20	50	100	200
S, N-GQDs-1	362.60	311.78	263.20	213.76	166.67	132.97
S, N-GQDs-2	264.30	212.02	167.45	155.18	124.96	110.12
S, N-GQDs-3	182.84	167.36	153.05	145.64	101.55	88.19
GQDs	144.37	131.63	120.27	109.66	98.84	78.69

**Table 2:** Comparison of specific capacitance values of different nanomaterials

Materials	Electrolyte	Specific capacitance values (F g^−1^)	Reference
S, N-GQDs	2 M KOH	362.60	This work
N-GQD/cMOF-5	1 M H_2_SO_4_	294.1	[37]
CQD aerogel	1 M LiClO_4_	294.7	[54]
Graphene nanosheets	6 M KOH	233.1	[55]
N-doped graphene	1 M KOH	282	[56]
N-GQD@cZIF-8/CNT	1 M H_2_SO_4_	540	[52]
Interconnected mesoporous carbon sheets	6 M KOH	242	[57]
Mn_3_O_4_-graphene QDs	6 M KOH	452.72	[58]
PVA-GQD/PEDOT	1 M H_2_SO_4_	291.86	[59]
NrGO/GQDs	6 M KOH	344	[60]
GQDs-graphene	6 M KOH	296	[61]
N-GQD@Fe_3_O_4_-HNTs	1 M Na_2_SO_4_	418	[62]
GQD-HNTs	1 M Na_2_SO_4_	363	[63]
Fe_2_O_3_ QDs/FGS	1 M Na_2_SO_4_	347	[64]
GQDs–3D graphene	1 M H_2_SO_4_	268	[65]
N-graphene nanosheet	6 M KOH	244.6	[66]
3D N-doped graphene	6 M KOH	334	[67]

## Conclusions

In summary, a top-down hydrothermal method was used to synthesize S, N-GQDs. The obtained S, N-GQDs present about 1–2 layers of graphene and well-defined lattice fringes with the interlamellar spacing of 0.34 nm ascribed to the (002) crystal face of graphene. Besides, the incorporation of S, N presents an absorption peak of S, N-GQDs around 405 nm, and exhibit an adjustable fluorescence characteristic in the excitation-visible range. Meanwhile, S, N-GQDs present remarkable capacitive performances due to the additional high pseudocapacitance provided by the doped S, N and the doping state acting as a trap state to enhance the charge storage capacity. S, N-GQDs-1 show excellent capacitive behavior and the specific capacitance is 362.60 F g^−1^ at a fixed scanning rate of 5 mV s^−1^. The ideal EDLC characteristics of S, N-GQDs confirms their new direction for the applications in energy storage devices.

## Data Availability

All data generated or analyzed during this study are included in this published article.

## Abbreviations

AFM: Atomic force microscope
CV: Cyclic voltammetry
EDLC: Electric double-layer capacitance
FT-IR: Fourier transform infrared spectroscopy
HRTEM: High-resolution transmission electron microscope
PL: Photoluminescence
S, N-GQDs: Sulfur, nitrogen co-doped graphene quantum dots
XPS: X-ray photoelectron spectroscopy
